# Deep Learning-Based Cell Detection and Extraction in Thin Blood Smears for Malaria Diagnosis

**DOI:** 10.1109/AIPR52630.2021.9762109

**Published:** 2021-04-26

**Authors:** Deniz Kavzak Ufuktepe, Feng Yang, Yasmin M. Kassim, Hang Yu, Richard J. Maude, Kannappan Palaniappan, Stefan Jaeger

**Affiliations:** * Department of Computer Science, University of Missouri-Columbia, MO, USA; † National Library of Medicine, National Institutes of Health, Bethesda, MD, USA; ‡ Mahidol Oxford Tropical Medicine Research Unit, Mahidol University, Bangkok, Thailand; § Centre for Tropical Medicine and Global Health, Nuffield Department of Medicine, University of Oxford, Oxford, UK; ¶ Harvard TH Chan School of Public Health, Harvard University, Boston, MA, USA

**Keywords:** Malaria diagnosis, *Plasmodium falciparum*, *Plasmodium vivax*, microscopy, thin blood smear, machine learning, image analysis

## Abstract

Malaria is a major health threat caused by Plasmodium parasites that infect the red blood cells. Two predominant types of Plasmodium parasites are *Plasmodium vivax* (*P*. *vivax*) and *Plasmodium falciparum* (*P*. *falciparum*). Diagnosis of malaria typically involves visual microscopy examination of blood smears for malaria parasites. This is a tedious, error-prone visual inspection task requiring microscopy expertise which is often lacking in resource-poor settings. To address these problems, attempts have been made in recent years to automate malaria diagnosis using machine learning approaches. Several challenges need to be met for a machine learning approach to be successful in malaria diagnosis. Microscopy images acquired at different sites often vary in color, contrast, and consistency caused by different smear preparation and staining methods. Moreover, touching and overlapping cells complicate the red blood cell detection process, which can lead to inaccurate blood cell counts and thus incorrect parasitemia calculations. In this work, we propose a red blood cell detection and extraction framework to enable processing and analysis of single cells for follow-up processes like counting infected cells or identifying parasite species in thin blood smears. This framework consists of two modules: a cell detection module and a cell extraction module. The cell detection module trains a modified Channel-wise Feature Pyramid Network for Medicine (CFPNet-M) deep learning network that takes the green channel of the image and the color-deconvolution processed image as inputs, and learns a truncated distance transform image of cell annotations. CFPNet-M is chosen due to its low resource requirements, while the distance transform allows achieving more accurate cell counts for dense cells. Once the cells are detected by the network, the cell extraction module is used to extract single cells from the original image and count the number of cells. Our preliminary results based on 193 patients (including 148 *P*. *Falciparum* infected patients, and 45 uninfected patients) show that our framework achieves cell count accuracy of 92.2%.

## I Introduction

Malaria remains a global health threat with considerable mortality rates. Diagnosis and monitoring of malaria have ongoing challenges particularly in resource-poor settings where experts analyzing the microscopy images are often lacking, and computational resources needed for automated analysis are limited. Light-weight automated systems that can assist health providers in diagnosis and monitoring of malaria disease is a critical need to ensure rapid and accurate diagnosis and treatment. Lately, thanks to the advances in computational resources and availability of large amounts of annotated data, supervised machine learning methods have started to be used for automated malaria diagnosis from thin blood smear microscopy images [1], [2]. However, there are still several problems that are needed to be addressed to develop a successful machine learning model that can segment and count the red blood cells in a thin blood smear microscopy image for malaria diagnosis. One of these problems is large appearance variations between the blood smear images. The smear images differ in color, contrast, and consistency due to different smear preparation and staining procedures. This variety brings generalization challenges making trained machine learning models harder to use on blood smears that are prepared at different locations. Another challenge is large numbers of touching or overlapping cells in thin blood smear images that lead to detection and segmentation problems. This is an important issue for malaria diagnosis and monitoring that require accurate counting of cells to calculate parasitemia (a measure of parasite load).

**Fig. 1:**
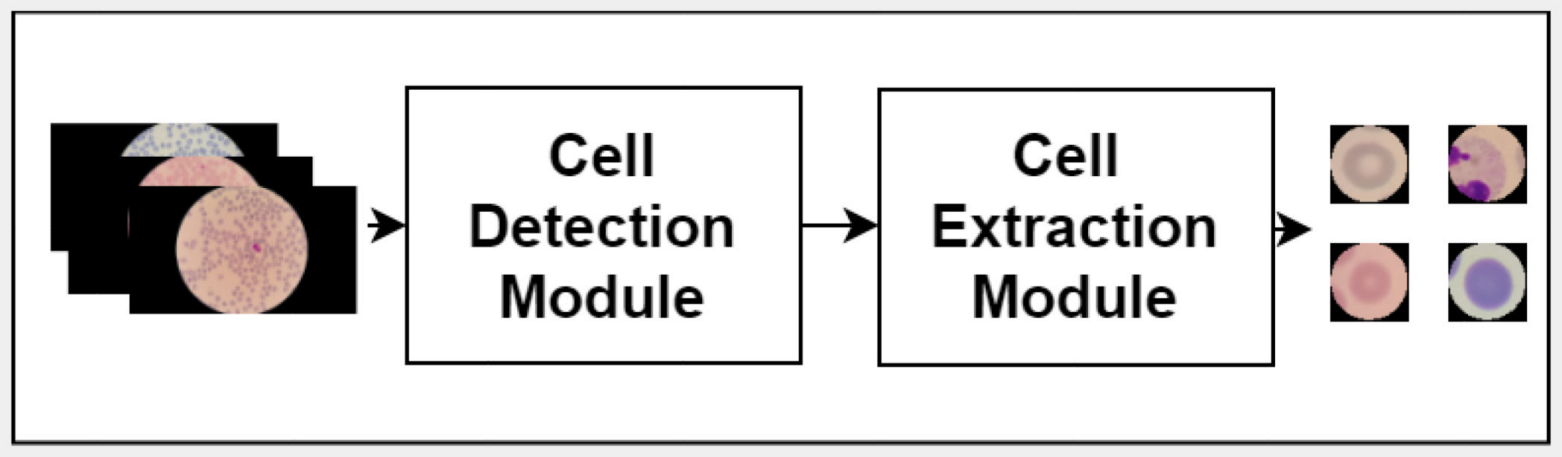
Flowchart of the proposed CFPNet-M-DEC framework.

In this paper, we present a pipeline called Channel-wise Feature Pyramid Network for Medicine (CFPNet-M) [3] - Detection, Extraction and Counting (CFPNet-M-DEC), to detect, extract and count red blood cells in thin blood smear microscopy images for automated malaria diagnosis and patient monitoring. To address the first problem caused by the appearance variations in collected blood smear images, we propose to use color deconvolution [4], [5], and the green channel of the image as inputs to the network. To address the second issue of distinguishing touching cells more accurately, the model is trained as a regression model rather than a binary segmentation model. The regression model learns a processed distance transform of the binary ground truth mask. As the segmentation network, CFPNet is used by modifying the first and the last layers to convert it to a regression network that takes a two channel input. CFPNet-M [3] is a light-weight network that is specifically developed for biomedical image segmentation. Due to its characteristics like the low memory requirement, it is suitable for resource-poor settings. The overall pipeline of the proposed red blood cell detection, extraction and monitoring system is given in Fig. 1. The original thin blood smear images are given as the input to the pipeline. These original images are processed to get the color deconvolution image and the green channel to be given to the cell detection module. The model in the detection module infers a truncated distance transform for each image, and the inferenced images are given to the cell extraction module. Finally, the cell extraction module uses a series of classical image processing techniques to extract each cell as a separate image and the cell count for further analysis.

## II Related Work

Automating malaria diagnosis and monitoring using thin blood smears is an active research area [2], [6], [7]. Thanks to the advances in deep learning and availability of annotated training data, recent works started to rely on supervised deep learning techniques. Some works directly use the whole microscopy image for tasks like segmentation and detection [8]–[13], some use extracted cell images for tasks like classification [11], [13]–[15]. Moreover, some works evaluate cell patches collected from multiple patients, and some others evaluate the results on patient level. There are different studies focusing on the detection of the red blood cells for further use [16], and the detection of the infected cells directly [1], [12], [17], [18]. Some studies classify red blood cells as infected vs. uninfected, as a two class problem [19], [20], and other studies approach the problem as a three or more class classification task and include classes to differentiate different species of parasites. Finally, there are studies that created a full pipeline for the detection and classification tasks [13], [21], [22]. On the other hand, the automated systems are often needed in environments with limited computational resources or lack of experts. In such environments, the most advanced tool on site might be a smart phone, which is successfully used in some recent works [23]–[26].

Like many other biomedical image analysis problems, there are problem specific issues coming from the nature of the data. Specifically, in malaria diagnosis, available data are collected from many different places, with varying image quality. Common challenges of image detection and classification in biomedical images include the variety of appearance of the same class cells, touching and partially overlapping cells [27]. Specifically, when accurate cell counts are important, as they are for malaria diagnosis and monitoring, touching and partially overlapping cells create even more critical issues. To address this problem, some authors used distance transform as ground truth for regression, instead of a binary mask in convolutional neural networks, to highlight the center of the cells or particles. This can lead to a better distinction of touching cells [28]–[30].

This study proposes a processing pipeline to detect, count, and extract red blood cells from thin blood smear images to be used for identification of infected versus uninfected cells and for classification of infecting parasite species. The proposed pipeline uses a distance transform-based cell segmentation approach to increase cell count accuracy, particularly in the presence of touching and overlapping cells. The proposed pipeline also involves a color deconvolution step [4], [5] to increase the generalization capability of the trained deep learning model to better adapt to processing of images collected from different sites.

## III Methods

We propose a framework, CFPNet-M-DEC, for detecting and extracting red blood cells in thin blood smears, as shown in Fig.1. It consists of two modules: cell detection module and cell extraction module. The cell detection module takes an original microscopy image as input and uses a modified CFPNet-M model to detect cells. Once the cells are detected by the network, the cell extraction module is used to extract and count single cells.

### A Detection Module

**Fig. 2:**
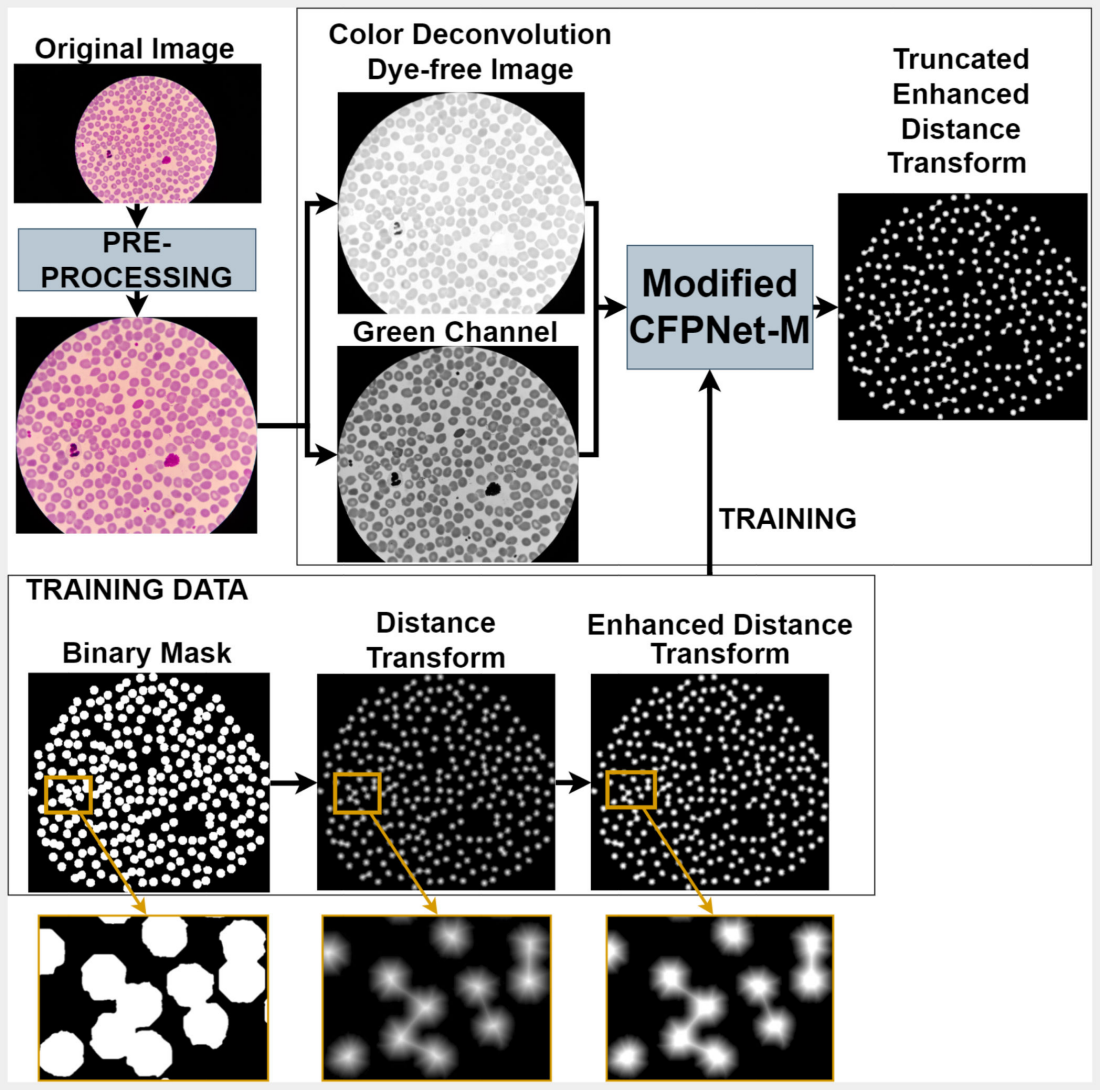
Different stages of the Cell Detection Module. The original image is pre-processed, then color deconvolution is done to get the dye-free image. The green channel is taken together with the dye-free image as the inputs to the modified CFPNet-M, which learns a truncated, enhanced distance transform.

The proposed detection module, illustrated in Fig. 2, includes three main steps: (i) image pre-processing, (ii) color deconvolution; and (iii) deep learning-based detection.

#### 1) Pre-processing

This step aims to detect and crop the circular image region seen through the microscope from the rest of the image. The original images (of size 5312 × 2988) include a dark background surrounding the blood smear image seen through the microscope. We used Otsu thresholding [31] to generate binary masks that differentiate blood smear regions from the surrounding background. Bounding boxes computed from these masks are then used to crop regions of interest from the original images. This step reduces image size to approximately 3000 × 3000. Cropped images and the corresponding ground truth segmentation masks are then resized to 800 × 800.

**Color Deconvolution**: Color deconvolution is an algorithm designed to extract the dyes of different stains from RGB images [4], [5]. Color deconvolution can be used to extract single or multiple stains from an image. The extraction of different dyes gives us a stain free, common color ground for a variety of images collected from different sources. Since the microscopy images are acquired from different labs and hospitals, the output image varies a lot in color and consistency. In the detection module, to exclude the unwanted effects of this variety, a color deconvolution image of the original microscopy image is used as one of the input channels. In our experiments, an extraction for Giemsa dye is done, but this method can be generalized to different dyes, therefore is applicable to other images prepared with different types of dyes.

#### 2) Training

To detect and extract individual cells for further analysis, we modified a recent light-weight segmentation network Channel-wise Feature Pyramid Network for Medicine (CFPNet-M) [3]. We modified the first and last layers of the network to allow processing of two channel (dye-free image and green channel of the original image) input; and to perform regression rather than classification. The network is trained to map its input to a processed distance transform of the binary segmentation mask as shown in Fig. 2. The CFPNet-M network [3] is an improved version of the classical U-Net [32] segmentation network. The number of trainable parameters of CFPNet-M is drastically less compared to the U-Net network, which reduces the resources needed for both training and inference phases. This property is important for malaria diagnosis in resource-poor settings. Adam optimizer and mean squared error loss function are used for training the network. The model is trained using the dye free image and the green channel of the original image as inputs, and truncated distance transform of the binary ground-truth cell segmentation mask as output. The training image is generated as follows: (i) a binary cell segmentation mask is created using manual polygon annotations generated for the cells in the input image; (ii) distance transform operation is applied to the binary segmentation mask; (iii) contrast enhancement is applied to the distance transform output to better highlight the cell centers; (iv) enhanced distance transform is truncated to a minimum distance to decrease the number of possible values for the regression process. The resulting truncated enhanced distance transform map is used to train the modified CFPNet-M network.

### B Cell Extraction Module

Once the truncated distance map of the cells is generated by the modified CFPNet-M network, post-processing steps are performed to extract individual cells for further analysis (i.e. infected vs. not infected classification). Given the network output, single cell image patches are extracted using the following steps: (i) A binarized version of the network output is used to remove background noise from the enhanced distance map; (ii) Gaussian smoothing is applied to the enhanced distance map to reduce false detections; (iii) extended-maxima transform [33] is applied to the smoothed distance map to detect inner cell regions; (iv) morphological erosion operation is applied to the extended-maxima map to reduce merging of neighboring cells; (v) individual cells are identified using connected component labeling; (vi) the labeled image is resized back to the original resolution (3000 × 3000 pixels); (vii) Fixed sized (200 × 200 pixels) image patches are extracted around the centroids of the connected components from the original resolution images.

## IV Experimental Results and Discussion

**Fig. 3:**
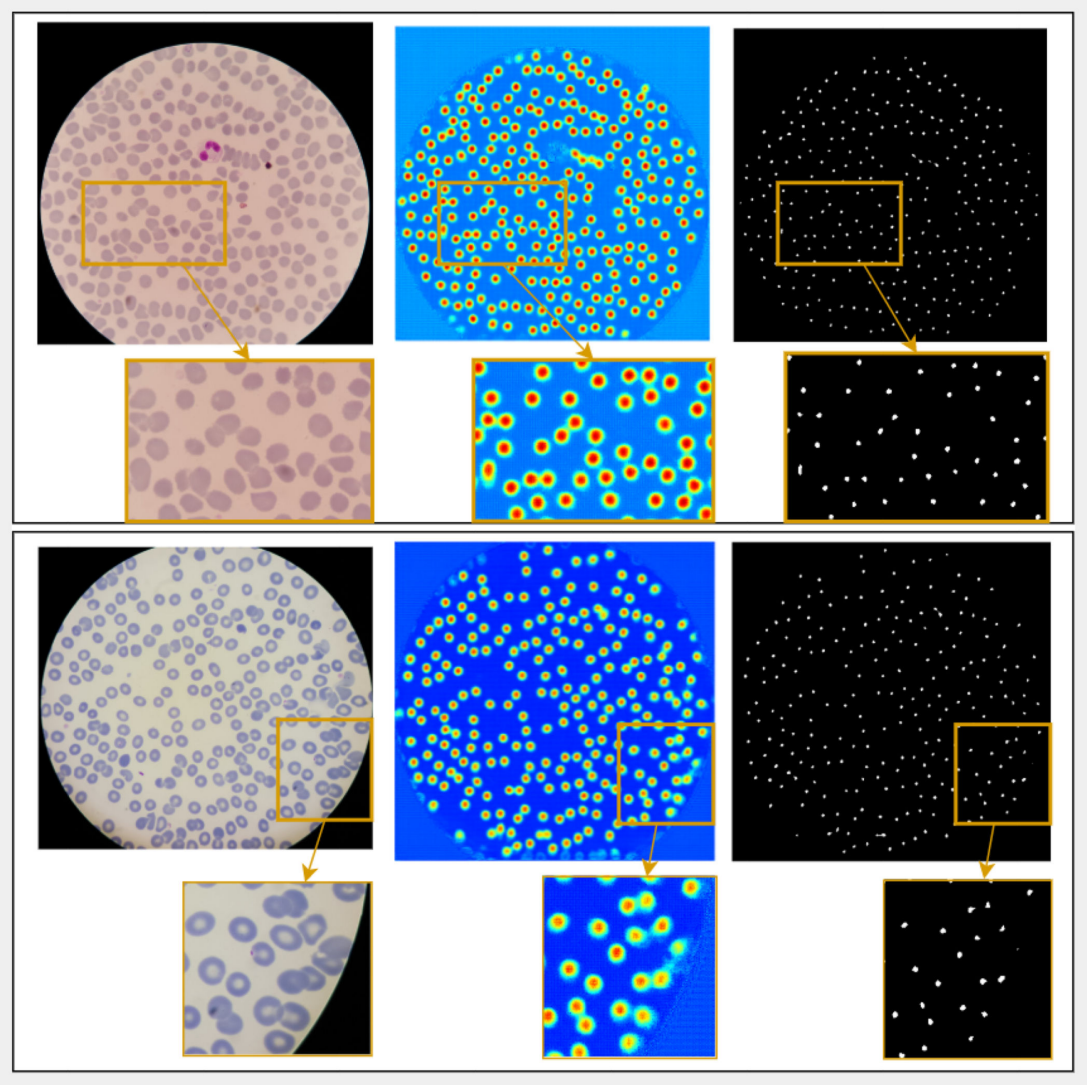
Example results from the CFPNet-M-DEC pipeline. Two example images are given in two rows with zoomed in parts showing touching cell examples. First column is the original image, second column is the model output shown in jet colormap, third column shows the cell center markers found in the model output.

The proposed system was trained, tested, and evaluated using data from 193 patients (including 148 *P*. *Falciparum* infected patients, and 45 uninfected patients). Out of 955 microscopy images in total, 100 images were selected for the validation set, 100 images were selected for the test set, and the remaining 755 images were used for training. Training was done using the default parameters of CFPNet-M, and for 25 epochs. Sample input images, corresponding inference results, and cell center markers obtained from these outputs are shown in Fig. 3. Separate cells are extracted using the markers as described in Section III-B. The results were evaluated in terms of segmentation and detection performances and compared to a marker controlled watershed segmentation algorithm [24].

### A Segmentation Performance

Experimental results were first evaluated in terms of cell segmentation. Model inference results were thresholded and dilated to produce binary segmentation masks and compared to the manual cell segmentation in terms of dice similarity coefficient [34]. The dice similarity coefficient between two binary images A and B is given in Eq. (1), where |*A*| represents the cardinality of image A.

(1) $$Dice(A,B)=\frac{2\left|\left(A\cap B\right)\right|}{\left|A\right|+\left|B\right|}$$

These results were compared to the performance of a watershed segmentation algorithm [24]. The marker controlled watershed algorithm was applied on the gradient magnitude image, based on the cell markers that were found using multi-scale Laplacian of Gaussian. The dice coefficient was calculated for each test image, then the mean dice coefficient was taken by averaging the dice coefficients of all images. The mean dice coefficient on all 100 images in the test set is given in Table I. In this table, we see that the proposed detection model outputs have a mean dice value of 0.5762, whereas the mean dice coefficient of the watershed-based method is 0.7204.

The results of these experiments show that the watershed-based method is superior to the proposed detection model with respect to the mean dice score with a 0.15 difference. This result is expected since the watershed-based method directly aims to have a segmentation mask, whereas the proposed model aims to have a detection mask that will later be used to extract and count each cell separately. Moreover, since the proposed model outputs a truncated distance transform, it highlights the centers of the cells while giving a smaller mask for each cell to make it easier to separate them in cases of touching and overlapping cells.

### B Cell Counting Performance

Experimental results from the full processing pipeline including the cell extraction module were evaluated in terms of single cell detection and counting. Extracted cells and the obtained cell counts were compared to the cell counts obtained from the binary ground-truth segmentation masks and the aforementioned watershed transform-based segmentation masks. Cell count errors in the ground-truth segmentation masks are caused by touching or overlapping cells. Cell count errors in this case can be used as a measure of image complexity. These counts were compared to the ground-truth cell counts obtained from the manual polygon annotations.

Mean cell count errors and standard deviations (STD) for all 100 images of 20 patients in the test set are shown in Table II. The mean errors show that the proposed pipeline has a 15.08 mean error value with 11.52 standard deviation. The binary ground truth segmentation masks have a 41.09 mean error with 19.16 standard deviation. Finally, the counts extracted from the watershed segmentation results have a 32.31 mean error with 40.66 standard deviation value. The mean error values in this table are calculated by taking the difference between the computed cell counts and the actual cell counts in each image, and then by averaging over 100 test images.

The cell count percentages for both image level and patient level are given in the Table III. The cell count percentage for a single image was directly calculated by taking the number of the extracted cells from the image divided by the actual cell count in that specific image. Then the ratio value was multiplied by 100 to have percentage scale. Finally, the mean of these percentages were taken for all 100 images to find the mean cell count percentage on image level, and for 5 images for each patient on patient level. The mean percentage value of the correctly detected and counted cells of the proposed pipeline on all test images is 92.28%, whereas the percentage value is 79.54% for the count of the cells that are extracted from ground truth masks used in training, and 84.07% for the cell counts extracted from the watershed-based method.

For patient level analysis of 20 patients, the percentage of correct counts of our method varies between 99.18% and 77.95%, with a mean of 92.20%. For watershed, the variance goes much higher with percentage values between 101.48% (over-segmentation) and 30.32%, with a mean of 83.61%. For the binary ground-truth segmentation masks, the percentage values varies between 88.70% and 56.41%, with a mean of 79.38%.

The experimental results show that our pipeline has the smallest mean error value with the lowest standard deviation with respect to the missed cells in the cell count. Moreover, the results show that our pipeline has the highest accuracy in cell count values with higher mean percentage with least variance. Our method goes up to 99.18% accuracy for cell counts on patient level. The proposed method is more focused on detecting and extracting single cells, rather than having accurate segmentation masks.

## V Conclusion

The proposed pipeline shows promising results for extraction and counting of red blood cells from thin blood smear images. The proposed deep learning network considers stain and color differences between images collected from different resources, and generates a truncated distance map instead of a binary segmentation mask. This distance mapping enables detection, extraction, and counting of single cells within clusters. The extracted cells can then be used to classify the individual cells as uninfected or infected, or to train a 3-class classifier for uninfected cells and cells infected with *P*. *falciparum* and *P*.*Vivax*. With a classification step added to the pipeline, the whole pipeline can be used to diagnose malaria, and monitor malaria patients during their treatments.

Our future work will involve generalization of the proposed pipeline to images collected from different resources including P. vivax; augmentation of the training dataset with artificial data to further improve performance on touching and overlapping cells; and precise tuning of the parameters or creation of adaptive parameters to extract the cells.

## Figures and Tables

**Table I:** Mean Dice Similarity Coefficient between the ground truth mask used in training, and the binarized detection mask result of Cell Detection Module of CFPNET-M-DEC, and the results of the watershed-based method.

Method	Mean Dice Coefficient
CFPNet-M-DEC	0.5762
Watershed	0.7204

**Table II:** GT Mask: Mean error and STD between the cell count of the cells extracted from the ground truth mask and actual cell count. Watershed: The mean error and STD between the cell count of the cells extracted from the results of the watershed-based method and the actual cell count. CFPNet-M-DEC: The mean error with the corresponding STD between the cell count of the cells extracted from the CFPNet-M-DEC result and the actual cell count.

Method	Mean Error	Error STD
Binary Ground-truth Mask	41.09	19.16
Watershed	32.31	40.66
CFPNet-M-DEC	15.08	11.52

**Table III:** GT Mask: Mean percentage of the cell count of the cells extracted from the ground truth mask over the actual cell count. Watershed: Mean percentage of the cell count of the cells extracted from the watershed-based method result over the actual cell count. CFPNet-M-DEC: Mean percentage of the cell count of the cells extracted from the CFPNet-M-Dec result over the actual cell count.

Mean Count Percentage %		
Method	Image Level	Patient Level
Binary Ground-truth Mask	79.54	79.38
Watershed	84.07	83.61
CFPNet-M-DEC	92.28	92.20
